# Adolescent late-onset riboflavin-responsive multiple acyl-CoA dehydrogenase deficiency manifesting with severe multi-organ failure: a case report

**DOI:** 10.3389/fped.2025.1513288

**Published:** 2025-07-02

**Authors:** Yunhua Zhao, Zhichao Li, Lili Cui, Jun Chen, Wangtao Zhong

**Affiliations:** ^1^ Department of Neurology, Affiliated Hospital of Guangdong Medical University, Zhanjiang, China; ^2^ Guangdong Key Laboratory of Age-Related Cardiac and Cerebral Diseases, Institute of Neurology, Affiliated Hospital of Guangdong Medical University, Zhanjiang, China

**Keywords:** multiple acyl-CoA dehydrogenase deficiency (MADD), electron transport flavoprotein dehydrogenase (ETFDH), multi-organ failure, autosomal recessive inherited disease, riboflavin

## Abstract

**Background:**

Multiple acyl-CoA dehydrogenase deficiency (MADD) is a rare autosomal recessive disorder characterized by dysfunctional acyl-CoA dehydrogenases, leading to lipid accumulation in various tissues, including skeletal muscles, liver, and cardiac muscles, etc. Late-onset MADD presents with progressive muscular symptoms (muscle weakness, atrophy, and myalgia) and even multisystem disorders (metabolic encephalopathy, dilated cardiomyopathy, liver failure, acute kidney injury, respiratory failure, and cardiac arrest). Over the past decade, only one case of childhood late-onset MADD with severe multi-organ failure has been reported.

**Case presentation:**

We report a 15-year-old girl with worsening muscle weakness, atrophy, myalgia, hepatic insufficiency, respiratory failure and even cardiac arrest. Laboratory tests showed significantly elevated levels of creatine kinase MB isoenzyme (CK-MB) and lactate dehydrogenase (LDH). A weakly positive serum small ubiquitin-like modifier 1 activating enzyme (SAE1) antibody suggested antibody-negative polymyositis (PM), but serum acylcarnitine analysis indicated increased concentrations of various acylcarnitines, while urine organic acids was normal. Muscle biopsy revealed significant lipid deposition within muscle fibers pointing to the diagnosis of lipid storage myopathy (LSM). Genetic testing identified a homozygous c.250G>A (p.Ala84Thr) mutation in electron transfer flavoprotein dehydrogenase (ETFDH), inherited from her parents. Although this pathogenic mutation is known in MADD, it has not been associated with adolescent late-onset MADD with severe multi-organ failure. After riboflavin supplementation, the patient regained mobility without ventilator support, with no recurrence of myopathic symptoms upon follow-up.

**Conclusion:**

MADD is a rare but treatable disease and its diagnosis is challenging due to its high clinical heterogeneity. Therefore, based on clinical, biochemical and pathological findings, gene analysis is critical for accurate diagnosis and clinical intervention, as riboflavin supplementation has shown lifesaving therapeutic benefit even in adolescent late-onset MADD with severe multi-organ failure.

## Introduction

Multiple acyl-CoA dehydrogenase deficiency (MADD) is a rare autosomal recessive metabolic disorder primarily caused by defects in electron transfer flavoprotein (ETF) or ETF dehydrogenase (ETFDH). These defects impair electron transport from acyl-CoA dehydrogenases to the respiratory chain, hindering the oxidation of fatty acids and amino acids, disrupting adenosine triphosphate (ATP) synthesis, and leading to lipid accumulation in muscle. MADD presents with varied clinical manifestations, generally classified into a neonatal-onset form, often fatal, with (type I) or without (type II) (1, 2). In contrast to the neonatal-onset forms, late-onset MADD may begin in childhood, adolescence, or adulthood, with onset ages ranging from 2 to over 60 years, and typically shows more variable and milder symptoms (3). Late-onset MADD often manifests with muscular symptoms, including proximal muscle weakness, atrophy, and myalgia, along with extra-muscular symptoms that may affect multiple organ systems, including metabolic encephalopathy, dilated cardiomyopathy, liver failure, and acute kidney injury (2, 4–6). Severe cases are rare but can progress to life-threatening respiratory failure and cardiac arrest (2, 4, 5). Only one case of childhood late-onset riboflavin-responsive MADD (RR-MADD) with severe multi-organ failure has been reported over a ten-year period (5). Therefore, MADD diagnosis is challenging due to clinical heterogeneity ranging from chronic muscular symptoms to multisystem disorders (7, 8).

Given clinical heterogeneity, diagnosis is primarily established through biochemical analysis of urine organic acids (increased concentrations of glutaric, ethylmalonic, adipic, butyric, sebacic, suberic, and isovaleric acids, etc.) and serum acylcarnitines (high levels of short-, medium- and long-chain acylcarnitines) (9). Muscle biopsy findings of lipid deposition within muscle fibers may be helpful in the differential diagnosis of lipid metabolic myopathy (LSM) with other metabolic myopathy (MM) or myositis. Advances in diagnostic methods, high-throughput sequencing (HTS) of ETF and ETFDH played a critical role in accurate diagnosis of MADD. ETFDH mutations account for over 90% of MADD cases, particularly in late-onset MADD (10). Significant mutations in ETFDH associated with late-onset MADD include c.250G>A (p.A84T), c.770A>G (p.Y257C), and c.1227A>C (p.L409F) (1, 11). Mutations such as c.341G>A (p.R114H) and c.1484C>G (p.P495R) in ETFDH have been identified as pathogenic in childhood late-onset MADD with Reye syndrome and severe multi-organ failure (5). Riboflavin responsiveness is observed in 98.4% of late-onset MADD cases, yet riboflavin efficacy in severe adolescent and adult-onset MADD with severe multi-organ failure has not been reported. This is the first report of adolescent late-onset RR-MADD with severe multi-organ failure associated with the c.250G>A (p.A84T) mutation in ETFDH.

## Case presentation

A 15-year-old girl was admitted to our hospital with a three-month history of progressive muscle weakness, limb atrophy, myalgia, and four days of dyspnea after anorexia and fatigue with academic stress. Her past medical and family history was unremarkable. The initial diagnosis was “myopathy of unknown etiology” after visits to local clinics and hospitals, where she received routine laboratory testing and traditional Chinese medicine (TCM). Four days prior, muscle enzymes revealed elevated creatine kinase (CK) 19,932 U/L (normal range 2.0–5.0) and lactate dehydrogenase (LDH) 2,245 U/L (normal range 89-221). Blood gas analysis indicated hypoxemia, and she was treated with non-invasive mechanical ventilation, Coenzyme Q10 (CoQ10), and Vitamin B. She was discharged from the local hospital without significant improvement. Her myopathic symptoms worsened, eventually leading to life-threatening respiratory failure and cardiac arrest upon transfer to our facility. Following six minutes of cardiopulmonary resuscitation (CPR) in the neuro-intensive care unit (NICU), the patient's stabilized. Physical examination revealed wet rales in the lungs and hepatomegaly. Neurological examination showed head drop, trunk muscle atrophy (particularly paraspinal muscles), proximal muscle weakness, and myalgia, with a Medical Research Council (MRC) score of 2/5–3/5 in the upper limbs and 1/5–2/5 in the lower limbs. Deep tendon reflexes and muscle tone were significantly reduced in the limbs, while pathological reflexes were normal.

**Figure 1:**
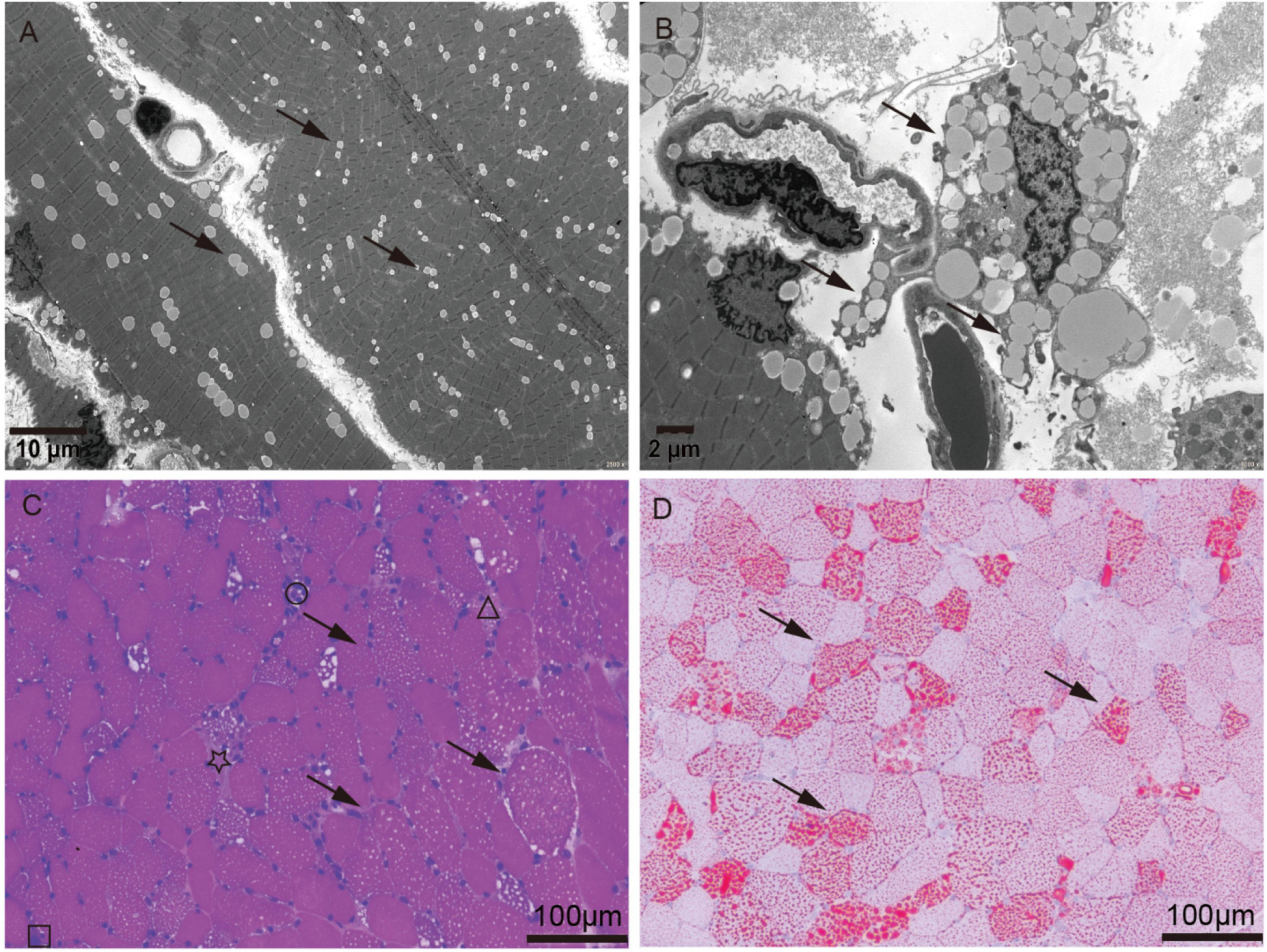
Muscle biopsy of quadriceps femoris. **(A,B)** Electron microscopy showed lipid deposits within muscle fibers (arrow). Scale bar, 10 mm for low-magnification images (2,500×) and 2 mm for high-magnification images (6,000×). **(C,D)** Hematoxylin and Eosin (HE) stain and Oil Red O (ORO) stain showed lipid deposits (arrow), atrophy (star), necrosis (triangle), regeneration (square), and lymphomonocytic infiltrating (round), in muscle fibers. Scale bar, 100 mm (20×).

**Figure 2:**
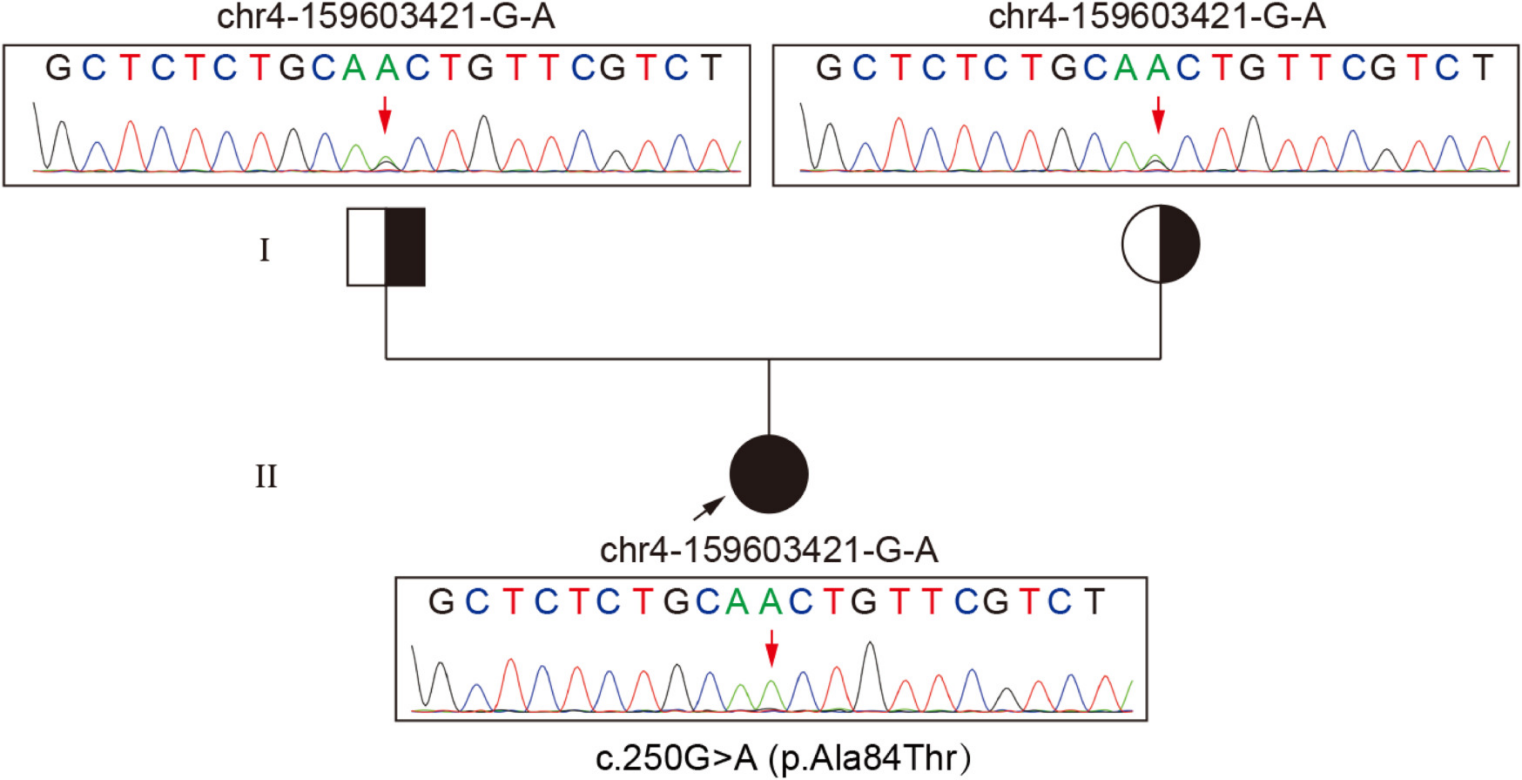
Chromatogram shows a homozygous c.250G>A substitution in ETFDH causing p.Ala84Thr and heterozygous parents.

Routine biochemical analysis showed marked elevations in muscle enzymes, including CK and LDH, as detailed in Supplementary Table S1. Chest imaging showed pulmonary infiltrates, increased cardiothoracic ratio, and emphysema in the neck and mediastinum. Abdominal imaging indicated hepatomegaly (Supplementary Figure S1). Electrocardiogram (ECG) indicated arrhythmia. Tranthoracic echocardiography (TTE) indicated left ventricular ejection fraction (LVEF) 55% (Supplementary Figure S2). Serum analysis showed a weakly positive small ubiquitin-like modifier 1 activating enzyme (SAE1) antibody (Supplementary Figure S2). Based on a suspected diagnosis of antibody-negative polymyositis (PM), empirical treatment with intravenous immunoglobulin (IVIg) and high-dose methylprednisolone was administered for 6 days. However, immunomodulatory therapy was discontinued due to lack of clinical improvement (Supplementary Figure S2). Given the severity and diagnostic uncertainty, and the suspicion of metabolic myopathy (MM) or inflammatory myopathies, biochemical analysis of serum acylcarnitine and urine organic acid was performed, as it was a useful and easily performed diagnostic tool. Urinary organic acid profile was normal, while serum acylcarnitine analysis indicated elevated levels of various acylcarnitine (Supplementary Table S1). Moreover, muscle biopsy and high-throughput sequencing (HTS) were performed with the patient's consent. Muscle biopsy of quadriceps femoris revealed significant lipid droplet accumulation in muscle tissue, without inflammatory lymphoplasmacytic infiltrates, glycogen deposits, ragged-red fibers (RRF), cytochrome c oxidase-negative (COX-negative) fibers or abnormal mitochondrial morphology (Figure 1). Genetic analysis revealed a homozygous c.250G>A (p.Ala84Thr) mutation in ETFDH, with Sanger sequencing confirming her parents as healthy carriers of this mutation (Figure 2). With the diagnosis of severe adolescent late-onset MADD, the patient was mainly treated with oral riboflavin (60 mg/day) for 40 days, along with Ubiquinol (60 mg/day), a low-fat and low-protein diet, rehabilitation exercises, and invasive mechanical ventilation, etc. (9, 12) (Supplementary Figure S2). On day 124, neuro-electrophysiological examination showed improvement in muscle weakness, with myogenic damage observed on electromyography (EMG) (Supplementary Figure S2). The patient was clinically stable and discharged on day 146 after regaining the ability to walk without ventilator support, following riboflavin supplementation. She remained free of recurrent myopathic symptoms and resumed normal daily activities with oral riboflavin (15 mg/day) by day 244.

## Discussion

Lipid storage myopathy (LSM) is a group of disorders characterized by impaired lipid oxidation, leading to the accumulation of lipid droplets in muscle fibers, resulting in muscle weakness or exercise intolerance (13). Since LSM was first reported in 1969, both familial and sporadic cases have increased (13). However, LSM remains a rare and heterogeneous disease that must be differentiated from other myopathies (14, 15). Initially, our patient was considered to have glycogen storage disease type II (Pompe disease) in the emergency room due to progressive skeletal muscle weakness, liver dysfunction, and later involvement of respiratory and cardiac muscles, which led to life-threatening respiratory failure and cardiac arrest following insufficient treatment at several local medical facilities (Supplementary Figure S2). The presence of myalgia upon further physical examination helped to exclude late-onset Pompe disease (LOPD), prompting differential diagnosis for polymyositis (PM). However, only a weakly positive small ubiquitin-like modifier 1 activating enzyme (SAE1) antibody was found in serum, which has a sensitivity of 100% and specificity of 99.6% for dermatomyositis (16). The patient did not present with the characteristic skin manifestations of dermatomyositis, aside from crystalline miliaria (Supplementary Figure S2), and myopathic symptoms and hepatic insufficiency, did not improve with immunotherapy. Given clinical manifestations heterogeneity, MADD diagnosis is primarily established through urine organic acid analysis and serum acylcarnitine analysis (7–9). Urinary organic acid analysis of patients with late-onset MADD is often very challenging, because elevation of urine organic acids may be subtle or elevated only during an acute metabolic crisis. These patients are prone to missed diagnosis and require a combination of serum acylcarnitines analysis (17). The girl's urinary organic acid profile was normal, while serum acylcarnitine analysis indicated increased concentrations of various acylcarnitines (Supplementary Table S1). Furthermore, muscle biopsy just revealed significant lipid deposition within muscle fibers without inflammatory lymphoplasmacytic infiltrates, glycogen deposits, ragged-red fibers (RRF), cytochrome c oxidase-negative (COX-negative) fibers or abnormal mitochondrial morphology pointing to the diagnosis of LSM rather than PM, glycogen storage disease type II (Pompe disease), mitochondrial myopathy (MM) or coenzyme Q10 deficiency.

There are four main causes of LSM: neutral lipid storage disease with myopathy (NLSDM), primary carnitine deficiency (PCD), neutral lipid storage disease with ichthyosis (NLSDI), and multiple acyl-CoA dehydrogenase deficiency (MADD), which is the most common cause of LSM in China. LSM with a known etiology shows better therapeutic outcomes, as riboflavin-responsive MADD (RR-MADD) with ETFDH mutation and carnitine-responsive PCD with SLC22A5 mutation. However, for LSM of unknown etiology, such as NLSDM, no effective treatment is currently available (18). Therefore, genetic analysis is essential for accurate diagnosis and potential future gene-targeted therapies.

Biallelic MADD variants have been reported to alter TNF-α-dependent signaling pathways and vesicular trafficking, leading to a wide phenotypic range from neurological abnormalities to multisystem disorders (7). Additionally, the clinical phenotype and severity of MADD are thought to correlate with the ETF/ETFDH genotype, resulting in different levels of residual enzyme activity (19). In China, ETFDH deficiency is the primary cause of MADD and serves as a reliable screening marker, significantly enhancing genetic diagnosis for MADD (1). Mutation analysis identified a homozygous c.250G>A (p.Ala84Thr) mutation in ETFDH, a single missense mutation associated with the patient's clinical presentation (1), indicating an adolescent late-onset MADD. Why was this case severe? It has been reported that ETFDH deficiency, riboflavin deficiency, and environmental or metabolic stresses (such as cold, infection, fatigue, or hunger) may together contribute to the pathogenesis of late-onset MADD. Therefore, it is proposed that on the basis of lipid metabolic defects due to the c.250G>A (p.Ala84Thr) mutation in ETFDH, anorexia and immune dysfunction after academic stress and fatigue, together with delayed medical care and pulmonary infection may have exacerbated the MADD, leading to progressive muscular symptoms, and even multi-organ failure. Furthermore, muscle biopsy revealed muscle fiber atrophy, necrosis, regeneration, and lymphomonocytic infiltration in muscle fibers, supporting a diagnosis of severe adolescent late-onset MADD (Figure 1).

Riboflavin supplementation is known to significantly improve myopathic symptoms and metabolic profiles in the vast majority of MADD patients, a condition known as riboflavin-responsive MADD (RR-MADD). Late-onset MADD is highly responsive to riboflavin, with a response rate of approximately 98.4%. Only one case of childhood late-onset RR-MADD with severe multi-organ failure has been reported in the past decade (5). Fortunately, this patient also showed a favorable clinical response to riboflavin replacement therapy, confirming a diagnosis of RR-MADD, despite adolescent late-onset MADD with multiple organ failure.

It was reported that the molecular mechanism of ribofavin treatment's signifcantly efficacy in MADD patients has been linked to its putative chaperone effects that can offset against inherited folding defects of ETFDH (20). Riboflavin supplementation may also increase cellular FAD (flavin adenine dinucleotide) levels and the binding between FAD and ETFDH to prevent protein degradation by stabilizing ETFDH and enhancing its concentration (21). Although most MADD patients are RR-MADD, understanding the molecular mechanism by which riboflavin corrects lipid metabolism disorders and alleviates clinical symptoms without altering the genetic defect remains an important area of research (9). Similarly, functional studies on mutation hotspots in RR-MADD are critical for future studies. Due to clinical heterogeneity and limited cases with comprehensive biochemical and gene diagnoses, a clear genotype-phenotype correlation has not been established. Thus, accurate diagnosis and treatment of this rare, pleiotropic disease remain challenging.

## Conclusion

MADD is a rare but treatable disease and its diagnosis is challenging due to its high clinical heterogeneity. Therefore, based on clinical, biochemical and pathological findings, gene analysis is critical for accurate diagnosis and clinical intervention, as riboflavin supplementation has shown lifesaving therapeutic benefit even in adolescent late-onset MADD with severe multi-organ failure.

## Data Availability

The datasets presented in this article are not readily available because of ethical and privacy restrictions. Requests to access the datasets should be directed to the corresponding authors.
