# Supplementary material for: Adolescent late-onset riboflavin-responsive multiple acyl-CoA dehydrogenase deficiency manifesting with severe multi-organ failure: a case report
Source: Front Pediatr. 2025 Jul 2;13:1513288. doi: 10.3389/fped.2025.1513288 (PMC12263563; doi:10.3389/fped.2025.1513288)
Supplement: Supplementary file 1 [file Datasheet1.pdf]

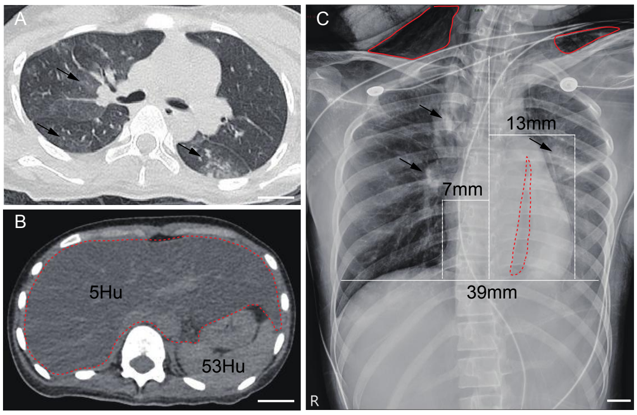

Figure S1. (A) CT scan of the lung. The scan showing patchy and ground glass high density shadows in both lungs (arrow). Scale bar, 2.5cm. (B) CT scan of the abdomen. The scan showing an enlarged liver with the liver/spleen CT ratio less than 0.5 (red dashed line). Scale bar, 2.5 cm. (C) X-ray chest radiograph shows an increased cardiothoracic ratio (more than 0.5), scattered patchy and striped shadows in both lungs (arrow), banded low-density shadow in the left edge of the heart, and subcutaneous emphysema in the neck (red dashed line). Scale bar, 2.5cm.

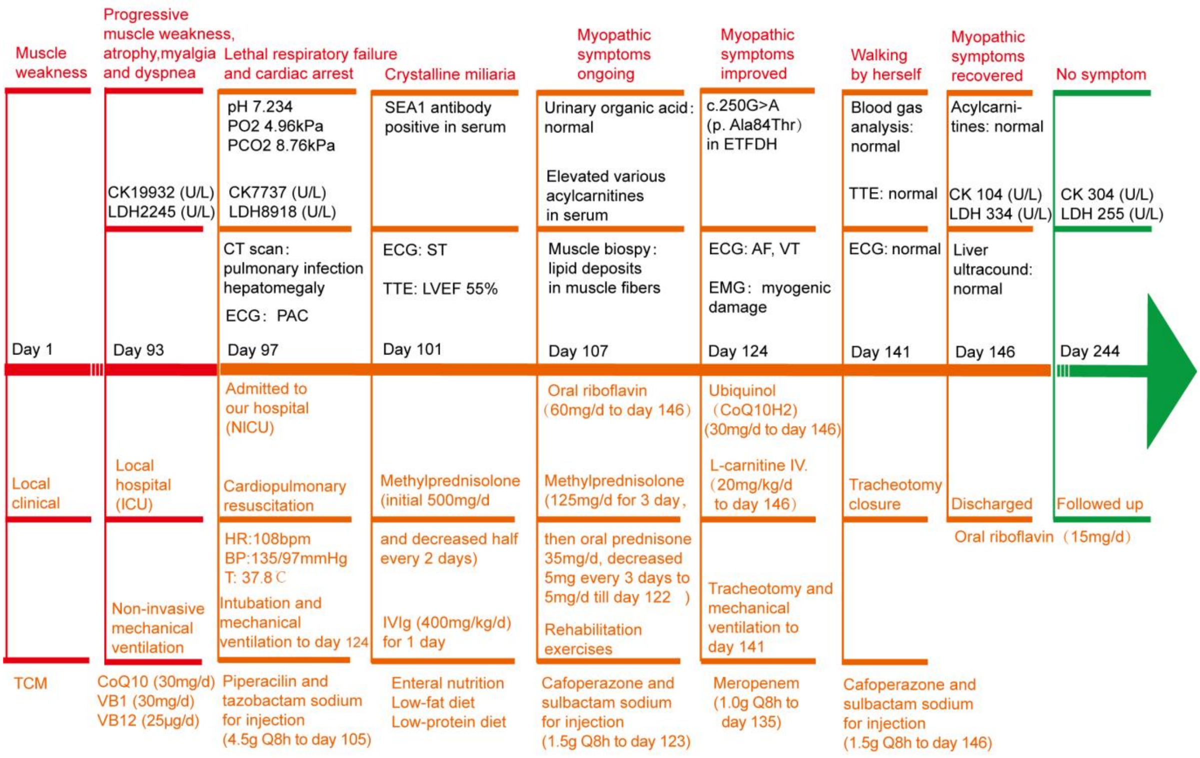

Figure S2

Figure S2. Timeline with clinical manifestation, treatment progression, and diagnosis time. TCM, traditional Chinese medicine; CK, creatine kinase; LDH, lactate dehydrogenase; ICU, Intensive Care Unit; NICU, Neuro Intensive Care Unit; HR, heart rate; BP, blood pressure; T, temperature; ECG, electrocardiogram; CT, computed tomography; Coenzyme Q10 (CoQ10); VB1, Vitamin B1; VB12, Vitamin B12; SAE1 antibody, small ubiquitin-like modifier 1 activating enzyme antibody; IVIg, intravenous immuno-globulins; PAC, premature atrial contraction; ST, sinus tachycardia; LVEF, left ventricular ejection fraction; TTE, tranthoracic echocardiography; AF, atrial fibrillation; VT, ventricular tachycardia; ETFDH, electron-transferring-flavoprotein dehydrogenase; EMG, electromyography.

Table S1 Outlines of the patient and results of routine laboratory tests.

	Day 97	Day 146	Day 244	Reference value[a]
**Blood Cell Count**				
White blood cell (WBC)	8.43	12.91 ↑	8.86	4-10*10^9/L
Neutrophils% (NE)	77 ↑	88.3 ↑	48.8 ↓	50-70%
Red blood cell (RBC)	3.63 ↓	3.98 ↓	4.72	4-5.5*10^{12}/L
Hemoglobin (HGB)	108 ↓	121	137	110-150g/L
**Blood Chemistry**				
Creatine kinase (CK)	7737 ↑	104	304 ↑	25.0 – 170.0U/L
Creatine kinase MB isoenzyme (CK-MB)	261 ↑	25.4 ↑	30.5 ↑	2.0 – 25.0U/L
High-sensitive troponin-T (hs-cTnT)	0.052 ↑	0.104 ↑	-	0-0.014ng/mL
N-terminal pro-B-Type natriuretic peptide (NT-proBNP)	881 ↑	36.8	-	<125pg/mL
Alanine aminotransferase (ALT)	2314 ↑	21.1	20.8	7.0-40.0U/L
Aspartate aminotransferase (AST)	2211 ↑	30.9	27.8	9-48U/L
Lactate dehydrogenase (LDH)	8918 ↑	334 ↑	255 ↑	89-221U/L
α -hydroxybutyrate dehydrogenase (α -HBDH)	7953 ↑	284.8 ↑	186.8 ↑	72.0-182.0U/L
Sodium ion (Na^+)	151 ↑	141	141	136-146mmol/L
Total protein (TP)	56 ↓	59.6 ↓	74.8	60-82g/L
Prealbumin (PAB)	78 ↓	235.5	283	155-400g/L
Total cholesterol (TC)	1.68 ↓	4.88	3.78	3.1-5.7mmol/L
Triglycerides (TG)	9.99 ↑	3.4 ↑	1.69	0.4-2.0mmol/L
Low-density lipoprotein (L-LDL)	0.66 ↓	2.3	1.78 ↓	1.8-3.36mmol/L
Creatinine (Cr)	17 ↓	17 ↓	31 ↓	34-80 μ mol/L
Urea nitrogen (BUN)	2.3 ↓	1.97	2.33 ↓	2.76-8.07mmol/L
Potassium (K^+)	3.31 ↓	3.56	4.45	3.5-5.5mmol/L
Blood glucose (Glu)	8.05 ↑	4.12	5.88	3.9-6.0mmol/L
**Blood gas analysis**				
pH	7.234 ↓	7.417	-	7.35-7.45
PO_2	4.96 ↓	13.13	-	10.64-13.3kPa
pCO_2	8.76 ↑	5.05	-	4.26-5.99kPa
$HCO3^-$	27.1 ↑	23.9	-	21-27mmol/L
Lactate (lac)	4.42 ↑	2.22 ↑	-	0.5-1.7mmol/L

**Serum acylcarnitine (AC) analysis**				
Free carnitine (C0)	158.9 ↑	7.66 ↓	-	10-100 μ mol/L
Butyrylcarnitine (C4)	1.97 ↑	0.09	-	0.05-0.6 μ mol/L
Palmitoylcarnitine (C16)	3.99 ↑	0.61	-	0.2-3.5 μ mol/L
Palmitoleylcarnitine (C16:1)	0.39 ↑	0.09	-	0.02-0.3 μ mol/L
Octadecanoylcarnitine (C18)	2.3 ↑	0.34	-	0.1-1.5 μ mol/L
Octadecenoylcarnitine (C18:1)	4.01 ↑	0.83	-	0.2-2.8 μ mol/L

[a] The reference values used at Affiliated Hospital of Guangdong Medical University
